# Reflections on theoretical framework use in nursing research

**DOI:** 10.1590/0034-7167-2024-0486

**Published:** 2024-07-29

**Authors:** Maria Ribeiro Lacerda, Rudval Souza da Silva, Nadirlene Pereira Gomes, Silvana Regina Rossi Kissula Souza

**Affiliations:** I Universidade Federal do Paraná. Curitiba, Paraná, Brazil; II Universidade do Estado da Bahia. Senhor do Bonfim, Bahia, Brazil; III Universidade Federal da Bahia. Salvador, Bahia, Brazil

**Keywords:** Research, Science, Nursing Theory, Nursing Research, Research Personnel, Investigación, Ciencia, Teoría de Enfermería, Investigación en Enfermería, Investigadores

## Abstract

**Objectives:**

to reflect on theoretical framework use in nursing research.

**Methods:**

a theoretical-reflexive study, based on concepts and constructs pertinent to using nursing theories and other sciences, considering issues of epistemology or philosophy of science.

**Results:**

we presented what it is and why to do nursing research and what a theoretical framework is and why to use it, in addition to some considerations regarding theoretical framework use in nursing research, essential for constructing disciplinary knowledge, which enables the materialization of researchers’ work and the presentation of propositions resulting from investigations in and for nursing as a discipline and science.

**Final Considerations:**

based on a reflection based on epistemological conceptions, it is possible to affirm that a theoretical framework is the core of researchers’ thinking, delimiting a problem to be investigated and, based on it, outlining methodological strategies to be followed, supporting nursing action and thinking as discipline and science.

## RESUMEN

**Objetivos:**

reflexionar sobre el uso de referentes teóricos en la investigación en enfermería.

**Métodos:**

estudio teórico-reflexivo, basado en conceptos y constructos relevantes para el uso de las teorías de enfermería y otras ciencias, considerando cuestiones de epistemología o filosofía de la ciencia.

**Resultados:**

presentamos qué es y por qué hacer investigación en enfermería y qué es el marco teórico y por qué utilizarlo, además de algunas consideraciones sobre el uso de marcos teóricos en la investigación en enfermería, esencial para la construcción del conocimiento disciplinar, que posibilita la materialización del trabajo del investigador y la presentación de proposiciones resultantes de las investigaciones en y para la enfermería como disciplina y ciencia.

**Consideraciones Finales:**

a partir de una reflexión sustentada en concepciones epistemológicas, es posible afirmar que el marco teórico es el núcleo del pensamiento del investigador, al delimitar un problema a investigar y, con base en él, delinear las estrategias metodológicas a seguir, apoyando la acción y el pensamiento de la enfermería como disciplina y ciencia.

## INTRODUCTION

Nursing, as a disciplinary field of knowledge, has produced research on a national and international level. In the Brazilian scenario, this has occurred, specifically, due to an effort by the area in instances of institutional representations and professional education/training so that it is necessary for research results to impact these institutions as well as the practice of our professional field. As a consequence, there is nursing that responds to problems that require care by a nurse and team, which must be embodied in theoretical frameworks that make it possible to indicate best practices to individuals and communities.

It is known that, for such advances, in the field of science, nursing has expanded in graduate settings as a strategic space for knowledge production. In this regard, the search for improvement and theoretical-methodological rigor to be imprinted on the objects of investigations has been increasing, given that graduate studies are the privileged locus for knowledge production, thus assuming the research process centrality.

Thus, those who propose to produce in this strategic space are expected to comply with essential requirements for the advancement and improvement of nursing thinking and discipline. This process involves adopting epistemological, methodological, technical and practical resources and an academic-scientific stance that values and ensures fruitful and consistent research results in the construction of new knowledge and training of new researchers.

From the perspective of epistemological resources, there is a theoretical framework in research, which constitutes the structure that can sustain or support the theoretical contribution of a research study. The theoretical framework covers not only the theory, but the narrative explanation of how researchers use the theory and its underlying assumptions to investigate the research problem, making its use a necessary condition to substantiate the knowledge produced^(1)^.

Thinking about the responsibility of graduate studies in training researchers, this theoretical-reflexive essay is based on the authors’ concern (women and man), throughout their careers as researcher(s) in nursing, about theoretical framework use, considered the core in knowledge construction.

## OBJECTIVES

To reflect on theoretical framework use in nursing research.

## METHODS

This is a theoretical-reflective study, based on concepts and constructs pertinent to the use of nursing theories and other sciences, considering the philosophy of science and epistemology^(1)^. This reflection is part of a conference entitled “Theoretical framework in Nursing Research”, given by one of the authors during the 22^nd^ Brazilian National Nursing Research Seminar (SENPE - *Seminário Nacional de Pesquisa em Enfermagem*) and the 3^rd^ International Nursing Research Seminar (SEINPE - *Seminário Internacional de Pesquisa em Enfermagem*), which took place in 2023 in the city of Curitiba, Paraná, Brazil.

The presentation of arguments about theoretical framework use requires considerations about the purpose and relevance of research in and for nursing, structured as follows: what is and why do nursing research? What is a theoretical framework and why use it?

## DISCUSSION

### What is and why do nursing research?

We understand research as a process of investigation, interpretation and modernization of scientific knowledge, assuming it as a tool for the efficient construction of knowledge, in order to enable us to deconstruct old hypotheses and develop new facts that make scientific evidence viable^(2)^.

The specific purposes of health research and, in this essay, nursing, include identification, description, exploration (descriptive, qualitative, quantitative and mixed-methods research), explanation (interpretation studies), prediction and control (propositional and evaluative studies), in addition to innovation and technology development. In terms of nature, basic research is carried out to expand the knowledge base in a discipline or to formulate or refine a theory while applied research focuses on finding solutions to existing problems^(3)^.

In addition to these, it is worth highlighting analytical research, carried out using rigorous and carefully designed procedures in the data collection and analysis process. In this type of research, the development of generalizations, principles and theories that can be useful in predicting future occurrences is emphasized, which requires using consistent theoretical frameworks^(4)^.

Regardless of the possibilities of doing research, all scientific investigation is essential for the advancement of any discipline. In nursing, it is no different, therefore, research can revolutionize it. In this regard, the Pan American Health Organization (PAHO) recognizes that research and production of knowledge in nursing constitute a strategic priority throughout the world^(5)^.

Promoting advances in nursing discipline and science is one of the great contributions of research, which aims to develop knowledge about health and nursing, in order to increase people’s skills in responding effectively to real or potential problems, whether for the care of themselves, others and/or communities. Such advances must evolve with the society’s needs and the progress of science so that research can contribute to shaping the professional field of nursing, considering care, teaching, research and management as it assists nurses and other professionals in evidence-based care^(6)^.

That said, it is up to us to reflect on: how research that has been produced is responding to communities’ problems and why this response occurs or not? At what level? A possible answer may be to rely on use or disuse of theoretical frameworks that would lead researchers to point out and situate problems and return research based on aspects that adhere to real situations.

At this point, it is worth highlighting and asking ourselves whether we have complied with what is stated in legislation regarding using theories, including in thinking about practice and, consequently, in research development, from the Federal Nursing Council (Cofen - *Conselho Federal de Enfermagem*) Resolution 736/2024, which determines, in its Article 2, the need and relevance that “Nursing Process must be based on theoretical support, which can be associated with each other, such as Care Theories and Models, Standardized Language Systems, validated risk prediction assessment instruments, Evidence-based protocols and other related knowledge, such as conceptual and operational theoretical frameworks that provide descriptive, explanatory, predictive and prescriptive properties on which they are based”^(7)^.

It is therefore inferred that nursing is a discipline that requires research skills, since decision-making requires knowledge and information that ensure responsible and effective care. To this end, nursing research has sought to build an advanced body of knowledge that aims to provide professionals with possibilities to provide efficient and cost-effective healthcare. It also shapes the attitude of nurses and other nursing team members, in a constant search for improvements in technical and relational skills, while filling gaps and contributing to the discovery of new knowledge about practice.

In academic training, research groups are constituted as training spaces for researchers, enabling the expansion of knowledge production^(8)^. Therefore, there is an urgent need for training to improve the skills needed to become researchers, which involves developing a broad and competitive profile capable of planning and managing scientific research projects, in addition to producing and implementing their results in order to cooperate with the progress of nursing and health knowledge.

Therefore, it is up to us to reflect and question: how has the experience of undergraduate and graduate students in research groups in research project management and execution been? In these spaces, have there been discussions about epistemological or philosophical issues to the point of promoting a real search for foundations in such perspectives? It is important to bring this provocation so that, during moments of discussions about theoretical-methodological resource selection, a look at the use of theories as theoretical support to broaden the vision of the phenomenon to be investigated is awakened.

### What is a theoretical framework and why use it?

A theoretical framework is a monocular strategy used by researchers to think about the problem and the path to be taken to answer it; it is the light to guide data analysis, in order to find pertinent and appropriate propositions for the practice of our professional field in all its areas of activity. Thus, using a theoretical structure aims to support researchers in clarifying epistemological dispositions, identifying the logic behind methodological choices and building a theory as a result of research, manifesting itself as a guide for study/investigation.

A theoretical framework is found at the intersection between three elements: existing knowledge and previously formed ideas about a given complex phenomenon; researchers’ epistemological dispositions in a kind of lens; and a methodically analytical approach^(1)^. Therefore, to define the framework to be used in research, a systematic study of scientific knowledge’s philosophical bases is necessary, enabling researchers to gain knowledge about the framework of basic references where theories and research assumptions are located.

In the meantime, it is important to point out the difference between theoretical framework and literature review, mistakenly considered as synonyms. The literature review, with a more restricted and specific purpose, is the structure that logically shows how the object or phenomenon of study is presented in the context of literature; it is like a narrative review of the state of the art regarding the research problem, the phenomenon to be investigated, including pointing out the knowledge gap to be filled.

Thus, research training needs to consider that the different methodological paths need to be followed with faithful observance of the philosophical assumptions that guide them. Knowledge of these assumptions, implicit in theoretical framework and explicit in the dialogue that each researcher, in particular, carries out when problematizing their research topic, enables them to create different research designs, in a creative freedom responsible for safeguarding coherence with the philosophical pillars^(9)^. It is in this act of constant vigilance that scientific rigor lies.

When considering theoretical framework applicability, it is often observed that it has not been used in the development of research with a quantitative approach, its use being restricted to qualitative studies^(6)^, and we need to ask ourselves why this only occurs in this type of study. It is urgent to train researchers that promote understanding of the need for a theoretical framework in nursing research, regardless of the methodological approach, be it quantitative, qualitative or mixed-methods, in order to support professional practice that positively impacts the healthcare of persons, families and communities.

Considering the clarification of the need to use a theoretical framework, the field of nursing knowledge has its foundations in the human, social and biological sciences. However, the foundation that these sciences can provide is not ready and does not respond to all the objects of study in our area, especially professional nursing care. Hence, it cannot be used in a prescriptive way in the daily practice of assistance and care. Thus, specific problems relating to nursing care need to be investigated when preparing empirical data in the light of theoretical frameworks, reconstructing them in order to provide a structure of knowledge that supports their practice^(9)^.

This process will provide a broad and focused view of the way of thinking that defines researchers and is constructed from their historical-social path, helping them to code and interpret their data. Therefore, it is important to emphasize that epistemological and ontological dispositions represent the architecture of how researchers see the world and sustain their knowledge production^(1)^. It is necessary to have mastery of this knowledge base from its origin, use and how it combines with knowledge in nursing to consolidate it and, thus, be able to appear as the substrate of the project, report and research products.

Consequently, consideration is necessary, and it is important to reflect: how should educators/advisors of graduate courses conduct training regarding theoretical framework use? Have these been used in the development of dissertations and theses? When they are actually adopted, have they expanded and improved the concepts and constructs arising from the frameworks assumed in each dissertation and thesis report?

It is known that researchers working in healthcare professions education are often advised to address and integrate the following concepts: theory and/or theoretical framework. Therefore, we need to clarify this differentiation. A theory is a set of propositions that are logically related, expressing the relationships between several different constructs and propositions. In other words, a theory is an abstract description of the relationships between concepts that help us understand the world^(4)^. For instance, we can highlight the use of qualitative approaches, nursing, sociology and other theories for research; for quantitative studies, epidemiology; and for mixed-methods research, the grand theories of the different disciplines of knowledge.

In turn, a theoretical framework corresponds to a set of logically developed and connected concepts and premises - based on one or more theories - that a researcher creates to structure a study. Thus, a theoretical framework is a reflection of the work of a researcher who commits to using a theory in a given study^(4)^. It is worth mentioning that funding agencies, research funding bodies, value projects containing articulated theoretical frameworks, assessing whether the study and its conceptual structure are explicitly described and justified.

Having made this distinction, we must question whether we have used such elements in the construction of knowledge in the Brazilian research scenario, considering that studies^(10)^ indicate that, in addition to the little or non-existent validity and/or reliability of data collection measures, the lack of a theoretical structure is the reason most cited as justification for the editorial decision not to publish a manuscript, according to a text in the Journal of Science Teacher Education. This shows that a meager theoretical framework or its absence is an equally critical problem for manuscripts submitted to scientific journals.

**Figure 1:**
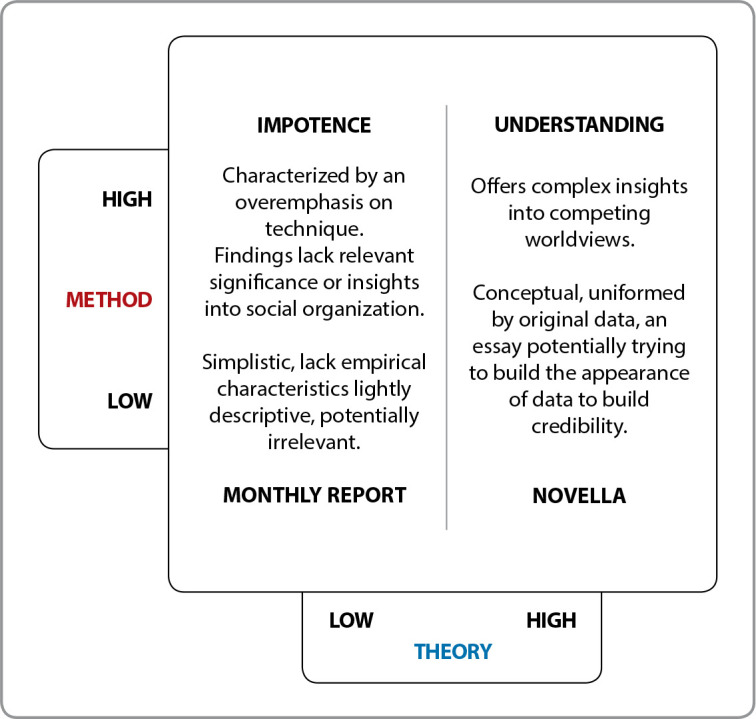
Evaluative quadrant highlighting the importance of method and theory^(1)^

Summing up, we reaffirm that a study with little theoretical basis and modest methodological resources ends up producing a simplistic report, running the risk of not being considered as knowledge production. Research with an excessive focus on theory and a poor method can be considered a theoretical essay, as it does not produce adequate integration between the original data and its analysis structure. On the other hand, excessive attention to the method without due attention to theory will produce an impotent study, little reflective and analytical, with excessive use of techniques that are not articulated. What is expected, in fact, is a study with high methodological rigor and theoretical framework, in order to produce complex knowledge and filled with competitive insights for the world of knowledge, as can be demonstrated in the infographic presented in Figure 1 ^(1)^.

Although much progress has been made since Nightingale’s time in terms of the amount of nursing research and diversity of topics studied, much of it has been carried out without an explicit conceptual or theoretical basis. However, we reinforce that theoretical structures are extremely important for all research work, in order to justify and validate the importance and meaning of the study^(10)^.

Gradually, nurses recognize that nursing research will have much more added value if it is guided by a theoretical framework or conceptual model rather than leaving it implicit^(11)^. Hence, the theoretical framework, which directs the object of study, starts from the use of a theory (or theories) in a study that simultaneously conveys the researchers’ deepest values and provides a clearly articulated indication or look at how the study will process new knowledge^(1)^.

In the graduate degree scenario, the appropriation of epistemological references is noticeable so that knowledge can be built in the scientific field, thus constituting an intrinsic requirement for candidate researchers applying for a place in graduate studies, especially doctoral candidates. Therefore, they must insert themselves, body and soul, into the contemporary epistemological debate, to become aware of the theoretical frameworks under which they intend to approach the sources of their research object^(3)^. From the debate, whatever its epistemological assumptions and technical-methodological mediations, there will always be a theoretical “interpretation” of empirical data, from which a meaning emerges.

Thus, science, as a modality of knowledge, only occurs as a result of the articulation of the logical with the real, the theoretical with the empirical. It is not reduced to a mere survey and presentation of facts or data collection; this is photography, a descriptive study, and we must evolve into explanatory, interpretative and propositional studies. These studies, yes, can be applied, (re)tested, indicated to support public policies and management processes and to support expenses and added values to nursing care, strengthening our professional field.

## FINAL CONSIDERATIONS

All thorough research, capable of absorbing knowledge and applicable to practice, has the theoretical framework as the substrate of its process. It is the core of researchers’ thinking when starting the path from thinking and delimiting the problem, based on reflections on epistemological concepts that indicate possibilities for professional nursing care congruent with what is necessary and what nursing can offer.

A theoretical framework corresponds to a magnifying glass from which researchers have the support to build their foundations for nursing discipline and science. It is what enables us to truly change our professional practice in a sustained manner, making us visible and empowered.
