# Supplementary material for: Reflections on theoretical framework use in nursing research
Source: Rev Bras Enferm. 2024 Jul 29;77(3):e20230486. doi: 10.1590/0034-7167-2024-0486 (PMC11290724; doi:10.1590/0034-7167-2024-0486)
Supplement: Translation

# Translation: Reflexões sobre o uso de referenciais teóricos nas pesquisas em enfermagem

**DOI:** 10.1590/0034-7167-2024-0486pt

**Authors:** Maria Ribeiro Lacerda, Rudval Souza da Silva, Nadirlene Pereira Gomes, Silvana Regina Rossi Kissula Souza

**Affiliations:** I Universidade Federal do Paraná. Curitiba, Paraná, Brasil; II Universidade do Estado da Bahia. Senhor do Bonfim, Bahia, Brasil; III Universidade Federal da Bahia. Salvador, Bahia, Brasil

**Keywords:** Pesquisa, Ciência, Teoria de Enfermagem, Pesquisa em Enfermagem, Pesquisadores

## Abstract

**Objetivos:**

refletir sobre o uso de referenciais teóricos nas pesquisas em enfermagem.

**Métodos:**

estudo teórico-reflexivo, fundamentado em conceitos e construtos pertinentes ao uso das teorias da enfermagem e outras ciências, considerando questões da epistemologia ou filosofia das ciências.

**Resultados:**

apresentamos o que é e por que fazer pesquisa em enfermagem, o que é o referencial teórico e por que utilizá-lo, além de ponderações concernentes ao uso de referenciais teóricos em enfermagem, essenciais para construção do conhecimento disciplinar, que possibilita a materialização do trabalho do pesquisador e a apresentação das proposições resultantes das investigações na e para a enfermagem enquanto ciência.

**Considerações Finais:**

a partir da reflexão pautada em concepções epistemológicas, é possível afirmar que o referencial teórico é o core do pensamento do pesquisador, ao delimitar um problema a ser investigado e, a partir dele, traçar estratégias metodológicas a serem seguidas, fundamentando o agir e pensar da enfermagem enquanto ciência.

## INTRODUÇÃO

A enfermagem, na qualidade de campo disciplinar do conhecimento, tem produzido pesquisas em âmbito nacional e internacional. No cenário brasileiro, isso tem ocorrido, especificamente, devido a um esforço da área nas instâncias de representações institucionais e de educação/formação profissional, de modo que se faz necessário que os resultados das pesquisas impactem essas instituições, bem como a prática de nosso campo profissional. Como consequência, tem-se uma enfermagem que responda aos problemas que necessitam dos cuidados da enfermeira e equipe, os quais devem estar consubstanciados em referenciais teóricos que possibilitem indicar melhores práticas aos indivíduos e coletividade.

Sabe-se que, para tais avanços, no campo da ciência, a enfermagem tem expandido, nos cenários da pós-graduação, como espaço estratégico para produção do conhecimento. Nesse sentido, vem aumentando a busca pelo aprimoramento e rigor teórico-metodológico a serem imprimidos nos objetos de investigações, haja vista ser a pós-graduação o lócus privilegiado para a produção de conhecimentos, assumindo, assim, a centralidade do processo de pesquisa.

Assim, daqueles que se propõem a produzir nesse espaço estratégico, espera-se o cumprimento de exigências imprescindíveis para o avanço e aprimoramento do pensamento e da disciplina da enfermagem. Tal processo perpassa pela adoção de recursos epistemológicos, metodológicos, técnicos, práticos e uma postura acadêmico-científica que valorize e assegure resultados de pesquisas profícuos e consistentes na construção de novos conhecimentos e na formação de novos pesquisadores.

Na perspectiva de recursos epistemológicos, há o referencial teórico nas pesquisas, que se configura a estrutura que pode sustentar ou apoiar o aporte da teoria de um estudo de investigação. O quadro teórico abrange não apenas a teoria, mas a explicação narrativa sobre como o pesquisador utiliza a teoria e seus pressupostos subjacentes para investigar o problema de pesquisa, tornando sua utilização uma condição necessária para substancializar os conhecimentos produzidos^(1)^.

Pensando na responsabilidade da pós-graduação na formação de pesquisadores, este ensaio teórico-reflexivo parte de uma inquietação das autoras e autor, ao longo de suas trajetórias como investigador(as) na área da enfermagem, sobre o uso de referenciais teóricos, considerados o cerne na construção do conhecimento.

## OBJETIVOS

Refletir sobre o uso de referenciais teóricos nas pesquisas em enfermagem.

## MÉTODOS

Estudo teórico-reflexivo, fundamentado em conceitos e construtos pertinentes ao uso das teorias de enfermagem e outras ciências, considerando a filosofia das ciências e a epistemologia^(1)^. Tal reflexão foi extraída da conferência “Referencial Teórico na Pesquisa em Enfermagem”, ministrada por uma das autoras durante o 22º Seminário Nacional de Pesquisa em Enfermagem (SENPE) e o 3º Seminário Internacional de Pesquisa em Enfermagem (SEINPE), que ocorreram no ano de 2023 na cidade de Curitiba, Paraná, Brasil.

A exposição de argumentos sobre o uso do referencial teórico requer considerações acerca do propósito e relevância da pesquisa em e para a enfermagem, sendo assim estruturada: o que é e por que fazer pesquisa em enfermagem? O que é o referencial teórico e por que utilizá-lo?

## DISCUSSÃO

### O que é e por que fazer pesquisa em enfermagem?

Entendemos pesquisa enquanto processo de investigação, interpretação e modernização do saber científico, assumindo-a como uma ferramenta para uma construção eficiente do conhecimento, de modo a nos possibilitar descontruir velhas hipóteses e desenvolver novos fatos que viabilizem evidências científicas^(2)^.

Os propósitos específicos da pesquisa em saúde e, neste ensaio, tratando-se da enfermagem, incluem a identificação, descrição, exploração (estudos descritivos, qualitativos, quantitativos e mistos), explicação (estudos de interpretação), predição e controle (estudos propositivos e avaliativos), além do desenvolvimento de inovação e tecnologia. No que tange à natureza, a pesquisa básica é realizada para ampliar a base de conhecimento em uma disciplina ou para formular ou refinar uma teoria enquanto a pesquisa aplicada se concentra em buscar por soluções para problemas existentes^(3)^.

Além dessas, vale destacar a pesquisa analítica, realizada a partir de procedimentos rigorosos e cuidadosamente elaborados no processo de coleta e análise de dados. Nessa modalidade de pesquisa, é enfatizado o desenvolvimento de generalizações, princípios e teorias que podem ser úteis na previsão de ocorrências futuras, o que requer o uso de referenciais teóricos consistentes^(4)^.

Independentemente das possibilidades do fazer pesquisa, toda investigação cientifica é essencial para o avanço de qualquer disciplina. Na enfermagem, não é diferente, logo, a pesquisa pode revolucioná-la. Nesse sentido, a Organização Pan-Americana da Saúde (OPAS) reconhece que a pesquisa e a produção do conhecimento na área da enfermagem constituem uma prioridade estratégica em todo o mundo^(5)^.

Promover avanços à disciplina e à ciência da enfermagem é uma das grandes contribuições da pesquisa, que visa desenvolver conhecimentos sobre saúde e enfermagem, a fim de aumentar as competências das pessoas em responder efetivamente aos problemas reais ou potenciais, seja para o cuidado de si mesmas, de outras e/ou da coletividade. Tais avanços devem evoluir com as necessidades da sociedade e o progresso da ciência, de tal modo que a pesquisa possa contribuir para moldar o campo profissional da enfermagem, considerando a assistência, o ensino, a pesquisa e a gestão à medida que auxilia enfermeiras e outras profissionais no cuidado baseado em evidências^(6)^.

Dito isso, cabe-nos provocar uma reflexão sobre: como as pesquisas que têm sido produzidas estão respondendo aos problemas da coletividade e por que essa resposta ocorre ou não? Em que nível? Uma possível resposta pode ser se sustentar no uso ou desuso de referenciais teóricos que levariam o pesquisador a pontuar e situar os problemas e o retorno das pesquisas diante de aspectos que tenham aderência a situações reais.

Nesse ponto, vale destacar e nos perguntar se temos atendido ao que está posto na legislação quanto ao uso das teorias, inclusive no pensar a prática e, consequentemente, no desenvolvimento de pesquisas, a partir da Resolução do Conselho Federal de Enfermagem (Cofen) nº 736/2024, que determina, no seu Art. 2º, a necessidade e relevância de que o “Processo de Enfermagem deve estar fundamentado em suporte teórico, que podem estar associados entre si, como Teorias e Modelos de Cuidado, Sistemas de Linguagens Padronizadas, instrumentos de avaliação de predição de risco validados, Protocolos baseados em evidências e outros conhecimentos correlatos, como estruturas teóricas conceituais e operacionais que fornecem propriedades descritivas, explicativas, preditivas e prescritivas que lhe servem de base”^(7)^.

Infere-se, pois, que a enfermagem é uma disciplina que exige habilidades de pesquisa, uma vez que as tomadas de decisões requerem conhecimentos e informações que assegurem o cuidado responsável e eficaz. Para isso, a pesquisa em enfermagem tem buscado construir um corpo avançado de conhecimentos que vise fornecer possibilidades ao profissional na prestação de cuidados de saúde eficientes e com boa relação custo-benefício. Igualmente, molda a atitude do enfermeiro e demais membros da equipe de enfermagem, em uma busca constante por melhorias nas habilidades técnicas e relacionais, ao mesmo tempo em que preenche lacunas e contribui para as descobertas de novos conhecimentos sobre a prática.

Na formação acadêmica, os grupos de pesquisa se constituem enquanto espaços formativos de investigadores, possibilitando a ampliação da produção do conhecimento^(8)^. Urge, assim, uma formação para o aprimoramento das habilidades necessárias para se tornarem pesquisadores, o que perpassa pelo desenvolvimento de um perfil amplo e competitivo capaz de planejar e gerenciar projetos de pesquisas científicas, além de produzir e empreender os seus resultados de modo a cooperar com o progresso do conhecimento da enfermagem e da saúde.

Destarte, cabe-nos refletir e questionar: como a vivência dos graduandos e pós-graduandos nos grupos de pesquisa no gerenciamento e na execução dos projetos de pesquisa tem se dado? Nesses espaços, tem havido discussões acerca de questões epistemológicas ou filosóficas a ponto de promover uma busca real por fundamentos em tais perspectivas? É importante trazermos essa provocação, para que, durante os momentos de discussões acerca da seleção dos recursos teóricos-metodológicos, seja despertado um olhar para com o uso das teorias como suporte teórico para ampliar a visão sobre o fenômeno a ser investigado.

### O que é o referencial teórico e por que utilizá-lo?

O referencial teórico é uma estratégia monocular utilizada pelo pesquisador para pensar o problema e o caminho a ser percorrido para respondê-lo; é a luz para guiar a análise dos dados, a fim de encontrar proposições pertinentes e apropriadas à prática de nosso campo profissional em todas as suas áreas de atuação. Dessa forma, o uso de uma estrutura teórica visa subsidiar o pesquisador no esclarecimento das disposições epistemológicas, na identificação da lógica por trás das escolhas metodológicas e na construção de uma teoria como resultado de pesquisa, manifestando-se enquanto guia para o estudo/investigação.

O referencial teórico encontra-se na interseção entre três elementos: o conhecimento existente e as ideias previamente formadas sobre dado fenômeno complexo; as disposições epistemológicas do pesquisador em uma espécie de lente; e uma abordagem metodicamente analítica^(1)^. Portanto, para definição do referencial a ser utilizado na pesquisa, faz-se necessário um estudo sistemático das bases filosóficas do conhecimento científico que possibilite ao investigador o conhecimento acerca do quadro de referências básicas onde situam-se as teorias e os pressupostos da pesquisa.

Nesse interim, importa pontuar a diferença entre referencial teórico e revisão de literatura, equivocadamente tidos como sinônimos. A revisão da literatura, com propósito mais restrito e específico, é a estrutura que mostra logicamente como o objeto ou fenômeno de estudo está apresentado no contexto da literatura; é como uma revisão narrativa do estado da arte acerca do problema de pesquisa, do fenômeno a ser investigado, inclusive apontando a lacuna do conhecimento a ser preenchida.

Assim, a formação em pesquisa precisa considerar que os diferentes caminhos metodológicos necessitam ser percorridos com fiel observância aos pressupostos filosóficos que os norteiam. O conhecimento desses pressupostos, implícitos no referencial teórico e explícitos no diálogo que cada pesquisador, em particular, realiza ao problematizar seu tema de investigação, possibilita-o à criação de diferentes delineamentos de investigação, em uma liberdade criadora responsável por resguardar a coerência com os pilares filosóficos^(9)^. É nesse ato de vigilância constante que reside o rigor científico.

Ao considerarmos a aplicabilidade do referencial teórico, por muitas vezes, observa-se que esse não vem sendo empregado no desenvolvimento de pesquisas de abordagem quantitativa, estando seu uso restrito aos estudos qualitativos^(6)^, e precisamos nos perguntar o motivo pelo qual isso só ocorre neste tipo de estudo. É premente uma formação de pesquisadores que promova a compreensão da necessidade de referencial teórico na pesquisa em enfermagem, independentemente da abordagem metodológica, seja ela quantitativa, qualitativa ou mista, a fim de subsidiar uma prática profissional que impacte positivamente o cuidado à saúde da pessoa, família e coletividade.

Considerando o aclaramento da necessidade do uso do referencial teórico, o campo de conhecimentos da área da enfermagem tem seus fundamentos situando-se nas ciências humanas, sociais e biológicas. Todavia, a fundamentação que essas ciências podem dar não está pronta e não responde a todos os objetos de estudos da nossa área, muito especialmente o cuidado profissional de enfermagem. Nesse sentido, não pode ser utilizada de forma prescritiva na prática cotidiana da assistência e do cuidar. Assim, os problemas específicos referentes ao cuidar da enfermagem precisam ser investigados, ao elaborar os dados empíricos à luz de referenciais teóricos, reconstruindo-os, de forma a possibilitar uma estrutura de conhecimentos que fundamente sua prática^(9)^.

Esse processo imprimirá um olhar amplo e direcionado pela forma de pensar que define o pesquisador e é construído a partir do seu percurso histórico-social, auxiliando-o na codificação e interpretação dos seus dados. Assim sendo, é importante ressaltar que as disposições epistemológicas e ontológicas representam a arquitetura de como o pesquisador vê o mundo e sustenta sua produção de conhecimento^(1)^. Há que ter domínio dessa base de conhecimento desde sua origem, utilização e como se aglutina aos conhecimentos da área da enfermagem para consolidá-lo e, assim, poder constar como substrato do projeto, relatório e produtos da pesquisa.

Consequentemente, uma ponderação se faz necessária, importando refletir: como os educadores/orientadores dos cursos de pós-graduação devem conduzir a formação relativa ao uso de referencial teórico? Esses têm sido utilizados no desenvolvimento das dissertações e teses? Quando são adotados de fato, têm ampliado e aprimorado os conceitos e construtos advindos dos referenciais assumidos em cada relatório de dissertação e tese?

Sabe-se que os pesquisadores que trabalham na educação de profissões na área da saúde são frequentemente aconselhados a abordar e integrar os seguintes conceitos: teoria e/ou quadro teórico. Logo, cabe-nos um esclarecimento acerca dessa diferenciação. Uma teoria é um conjunto de proposições que estão logicamente relacionadas, expressando as relações entre vários construtos e proposições diferentes. Em outras palavras, uma teoria é uma descrição abstrata das relações entre conceitos que nos ajudam a entender o mundo^(4)^. Exemplificando, podemos pontuar a utilização, para pesquisas de abordagens qualitativas, das teorias de enfermagem, sociologia e outras; para estudos de abordagens quantitativas, a epidemiologia; e para estudos mistos, as grandes teorias das distintas disciplinas do conhecimento.

Por sua vez, um quadro teórico corresponde a um conjunto de conceitos e premissas logicamente desenvolvidos e conectados - a partir de uma ou mais teorias - que um pesquisador cria para estruturar um estudo. Assim, um quadro teórico é o reflexo do trabalhar do pesquisador que se compromete a usar uma teoria em um determinado estudo^(4)^. Vale ressaltar que as agências de fomento, órgãos financiadores de pesquisas, valorizam projetos contendo quadros teóricos articulados, avaliando se o estudo e sua estrutura conceitual estão explicitamente descritos e justificados.

Feita tal distinção, cabe-nos questionar se temos usado tais elementos na construção de conhecimentos no cenário de pesquisa brasileiro, considerando que os estudos^(10)^ indicam que, além da pouca ou inexistente validade e/ou confiabilidade das medidas de coleta de dados, a falta de uma estrutura teórica é a razão mais citada como justificativa da decisão editorial de não publicar um manuscrito, segundo texto do *Journal of Science Teacher Education*. Isso mostra que uma parca estrutura teórica ou sua ausência é um problema igualmente crítico para manuscritos submetidos a periódicos científicos.

Em síntese, reafirmamos que um estudo com pouca base teórica e modestos recursos metodológicos acaba por produzir um relatório simplista, incorrendo no risco de não ser considerado como uma produção de conhecimento. Pesquisas com um foco em excesso na teoria e um método pobre podem ser consideradas um ensaio teórico, por não produzir uma adequada integração entre os dados originais e sua estrutura de análise. Por outro lado, o excesso de atenção ao método sem a devida atenção à teoria produzirá um estudo impotente, pouco reflexivo e analítico, com excessivo uso de técnicas que não se articulam. O que se espera, de fato, é um estudo com alto rigor metodológico e no referencial teórico, de modo a produzir conhecimentos complexos e regado de *insights* competitivos para o mundo do conhecimento, como pode ser demostrado no infográfico apresentado na Figura 1 ^(1)^.

**Figura 1:** Quadrante avaliativo com destaque para a importância do método e da teoria^(1)^

Embora muito progresso tenha sido feito desde a época de Nightingale em termos de quantidade de pesquisas em enfermagem e diversidade de temáticas estudadas, grande parte foi realizada sem uma base conceitual ou teórica explícita. Todavia, reforçamos que as estruturas teóricas são extremamente importantes para todo o trabalho de pesquisa, a fim de justificar e validar a importância e o significado do estudo^(10)^.

Gradualmente, as enfermeiras reconhecem que as pesquisas em enfermagem terão muito mais valor agregado se forem guiadas por um referencial teórico ou um modelo conceitual em vez de deixá-lo implícito^(11)^. Nesse sentido, o referencial teórico, que direciona o objeto de estudo, parte do uso de uma teoria (ou teorias) em um estudo que simultaneamente transmite os valores mais profundos do pesquisador e fornece uma indicação ou um olhar claramente articulada(o) de como o estudo processará novos conhecimentos^(1)^.

No cenário da pós-graduação, é perceptível a apropriação de referências epistemológicas para que se possa construir o conhecimento no campo científico, constituindo-se, assim, uma exigência intrínseca às candidatas a pesquisadores que pleiteiam uma vaga na pós-graduação, principalmente doutorandos. Portanto, impõe-lhe se inserir, de corpo e alma, no debate epistemológico contemporâneo, para se dar conta das referências teóricas sob as quais pretende abordar as fontes de seu objeto de pesquisa^(3)^. A partir do debate, quaisquer que sejam seus pressupostos epistemológicos e mediações técnico-metodológicas, existirá sempre uma “interpretação” teórica de dados empíricos, da qual germina uma significação.

Assim, a ciência, como modalidade de conhecimento, só se processa como resultado de articulação do lógico com o real, do teórico com o empírico. Não se reduz a um mero levantamento e exposição de fatos ou a uma coleção de dados; isso é fotografia, é estudo descritivo, e há que evoluir para estudos explicativos, interpretativos e propositivos. Esses estudos, sim, podem ser aplicados, (re)testados, indicados para subsidiar políticas públicas e processos gerenciais e para sustentar gastos e valores agregados aos cuidados de enfermagem, fortalecendo nosso campo profissional.

## CONSIDERAÇÕES FINAIS

Toda pesquisa criteriosa, capaz de absorver conhecimentos e aplicável à prática, tem o referencial teórico como o substrato do seu processo. É o core do pensamento do pesquisador ao iniciar o caminho a partir do pensar e delimitar o problema, pautado em reflexões acerca das concepções epistemológicas que indicam possibilidades para um cuidado profissional de enfermagem congruente com o que é necessário e com o que a enfermagem pode oferecer.

O referencial teórico corresponde a uma lupa a partir da qual o pesquisador tem o suporte para construir suas bases para a disciplina e ciência da enfermagem. É o que nos possibilita mudar realmente nossa prática profissional de forma sustentada, tornando-nos visíveis e empoderados.
